# Evaluation of Adaptive, Productive and Reproductive Performance of Boran Dairy Breed in the Lowland Agro‐Ecology of Kaffa Zone, South‐Western Ethiopia

**DOI:** 10.1002/vms3.71053

**Published:** 2026-06-23

**Authors:** Ephrem Worku Nigatu

**Affiliations:** ^1^ Department of Animal Science Wollega University Nekimte Ethiopia

**Keywords:** adaptation, biochemistry, Boran cattle, haematology, milk yield, reproductive traits

## Abstract

**Background:**

Adaptive traits, along with productive and reproductive performance, are essential for assessing the suitability and long‐term sustainability of indigenous cattle in specific production environments.

**Objective:**

This study evaluated the adaptive, productive and reproductive performance of Boran dairy cattle in the lowland agro‐ecology of the Kaffa Zone, south‐western Ethiopia.

**Methods:**

The study was conducted at a commercial farm in Gojeb, and sixty (60) multiparous Boran dairy cows were selected based on health status, parity, and lactation stage, following an adaptation period before data collection. This study covered the early, mid and late lactation stages over a 25‐week monitoring period. Daily milk yield was recorded twice daily, and reproductive parameters, including age at first calving (AFC), calving interval (CI), number of services per conception (NSPC), days open (DO) and gestation length (GL), were obtained from farm records. Adaptive performance was assessed using rectal temperature (RT) and respiration rate (RR) in relation to the meteorological variables. Blood samples were collected from 30 cows (10 per lactation stage) for haematological (HC, RBC, WBC and PCV) and biochemical (TP, GLU, URE, TGL, TC, AST and ALT) analyses. Data were analysed using SPSS version 20, and descriptive statistics were summarized as mean ± SD.

**Results:**

The current study revealed that the mean daily milk yield was 2.43 ± 0.62, 2.12 ± 0.58 and 1.72 ± 0.45 L/day for early, mid and late lactation, respectively. Parity 1 and 2 cows produced 1.71 ± 0.46 L and 2.47 ± 0.52 L, respectively, with an overall mean of 2.09 ± 0.62 L/day. Parity and lactation stage significantly (*p* < 0.001) affected production performance. The reproduction performance of Boran dairy cows was as follows: AFC (51.63 ± 1.82 months), CI (19.07 ± 1.93 months), GL (276.50 ± 2.15 days), DO (338.05 ± 21.71 days) and NSPC (1.60 ± 0.22). Lactation stage and parity significantly influenced several biochemical (TP, GLU, TGL, TC, ALT and AST) and haematological (HC, WBC and PCV) parameters, with total protein and urea being the main contributors to blood profile variation. Blood values remained within the normal range, indicating good physiological adaptation to the local conditions.

**Conclusion:**

The productive and reproductive performances of Boran cows were within the expected ranges for indigenous breeds, suggesting that crossbreeding could enhance productivity. Further studies should consider age, season and comparisons with other locally adapted breeds.

## Graphical abstract

Boran dairy cattle demonstrated acceptable productive, reproductive and adaptive performance under lowland conditions of south‐western Ethiopia. Significant effects of parity and lactation stage were observed on milk yield and blood parameters, while physiological values remained within normal ranges, confirming good adaptation and potential for sustainable dairy production.

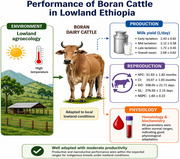

## 1 Introduction

### 1.1 Background of the Study

In Ethiopia, dairy production relies predominantly on indigenous livestock genetic resources, particularly cattle, which contribute to over 95% of the annual national milk yield (Dekebo and Kebede 2023). However, the national average milk yield per indigenous cow remains low at 1.48 L/day (Abegaz 2022). This low productivity is largely attributed to the limited genetic potential of local breeds, inadequate feed availability, high prevalence of diseases, poor animal health services and suboptimal husbandry practices (Vanvanhossou et al. 2021; Welay et al. 2018).

Ethiopia is home to approximately 37 indigenous cattle breeds, of which Arsi, Begait/Barca, Boran, Fogera, Horro, Sheko, Afar and Ogaden are well recognized and characterized (Hunde and Tadese 2020; Assefa et al. 2021; Asfaw et al. 2023). Among these, Fogera, Barca and Horro are primarily milk producers, whereas the remaining breeds are dual‐purpose, used for both milk and meat production (Mekonnen et al. 2022). The Boran cattle breed, categorized as *Bos indicus* (large‐humped East African Shorthorn Zebu type), is a dual‐purpose breed reared for milk and meat production (Galina and Geffroy 2023).

Boran cattle have evolved adaptive traits essential for survival in harsh environments. These include tolerance to periodic feed and water shortages, the ability to travel long distances in search of resources, efficient digestion of low‐quality feeds, heat stress tolerance and resistance to ticks, tick‐borne diseases and other tropical diseases (Mollong et al. 2025; Bayssa et al. 2021). In addition, Boran cattle are noted for their docility, high fertility and early maturation (Abdurehman 2019). Their adaptive mechanisms are mediated through a combination of morphological, behavioural, physiological, neuroendocrine, blood biochemical, metabolic, molecular and cellular responses that collectively promote survival and productivity under specific environmental conditions (Mollong et al. 2025; Getahun 2022).

Adaptation is influenced by genetic makeup and determines an animal's tolerance to adverse conditions. Heritable traits enhance the survival of populations and maintain genetic diversity, which facilitates matching breeds to changing environmental conditions while improving productivity and efficiency (Marchioretto et al. 2023). Therefore, evaluating the adaptive profile of cattle is essential for designing effective breed improvement strategies for indigenous and crossbred dairy cattle across diverse production systems (Pedlar et al. 2019).

Blood profiles provide valuable insights into the physiological adaptation, health status and metabolic efficiency of dairy cows during lactation. They are widely used to monitor herd health, detect subclinical diseases, identify metabolic disorders and evaluate nutritional adequacy (Barsila 2025). Haematological parameters, in particular, reflect an animal's adaptability to environmental stressors, whereas blood biochemical values within normal physiological ranges indicate good health and are closely associated with milk production (Karthik et al. 2021). Factors such as parity and lactation stage significantly influence haematological and biochemical parameters, as up to 80% of circulating metabolites are utilized by mammary secretory cells during lactation (Vallejo‐Timarán et al. 2020).

Several studies have evaluated the productive performance (milk yield) and reproductive performance, including age at first calving (AFC), days open (DO), gestation length (GL), and calving interval (CI) of Boran dairy cattle and their crosses in research stations, government farms and urban or peri‐urban dairy systems in Ethiopia (Endris 2017; Denbarga et al. 2012). However, these studies largely assessed production and reproduction in isolation, with limited consideration of how physiological adaptation influences milk yield and reproductive efficiency under different agro‐ecological conditions. Consequently, evidence linking adaptive indicators with milk productivity and key reproductive traits (AFC, DO, GL and CI) across lactation stages remains inadequate, particularly in lowland environments.

Boran heifers were introduced to the Gojeb Dairy Farm in the lowland agroecology of the Kaffa Zone in 2018 for milk production and crossbreeding. However, no location‐specific, integrated evaluation has been conducted to determine whether Boran cattle can simultaneously maintain normal adaptive status, achieve acceptable milk yield, and sustain efficient reproductive performance under these conditions. This lack of evidence constrains informed decisions regarding breed utilization, management, and crossbreeding strategies. Therefore, this study was designed to comprehensively evaluate the adaptive capacity, productive performance (milk yield), and reproductive performance of Boran dairy cattle in the lowland agroecology of the Kaffa Zone, Southwestern Ethiopia.

## 2 Materials and Methods

### 2.1 Study Area

**FIGURE 1:**
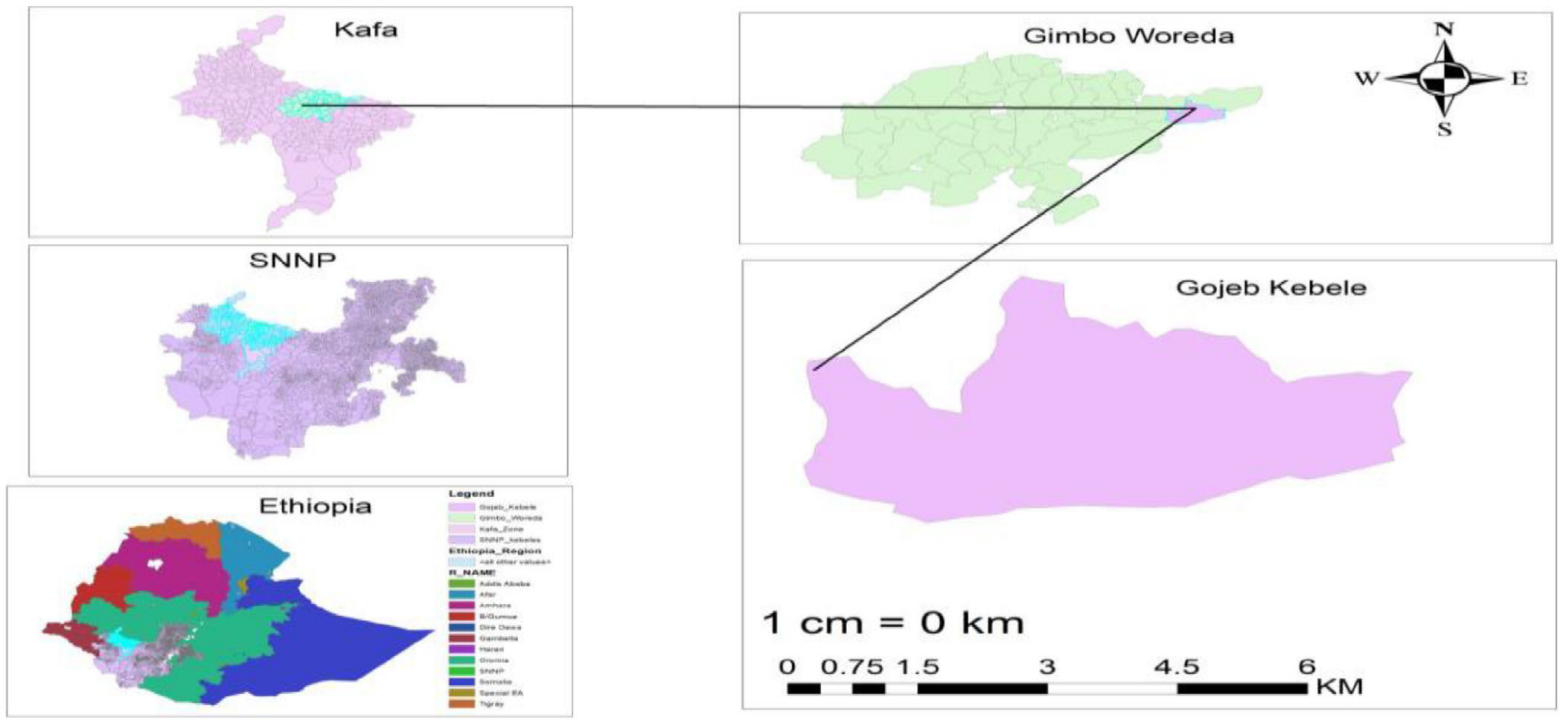
Location of the study area.

The study was conducted at the Midroc Investment Group agricultural farm in the Kaffa Zone, southwestern Ethiopia, near the border of Gimbo Woreda. The site is located approximately 75 km west of Jimma, 20 km north of Bonga and 531 km southwest of Addis Ababa at an altitude of 1305 m above sea level. The area receives an average annual rainfall of 1200 mm, with mean minimum and maximum temperatures of 15°C and 25°C, respectively (Figure 1).

### 2.2 Experimental Animal Management

The experimental animals were Boran cattle originally transported from the Borana Zone to the Gojeb Commercial Farm before the commencement of the study for milk production and crossbreeding purposes. Following the introduction, the animals were managed under standard management and environmental conditions of the farm. The study population consisted of animals that were subsequently born and raised on the farm, ensuring that all experimental animals had a long‐term exposure to the study environment.

Animals were selected based on their health status, parity and stage of lactation to ensure representative sampling across early, mid and late lactation. No separate control group was included; all comparisons were made within the study population using lactation stage and parity as internal reference groups. Lactating cows were uniformly managed according to farm standards. The animals were identified using ear tags and herd book records. The cows were stall‐fed natural pasture hay ad libitum and supplemented with 1 kg of concentrate feed before afternoon milking. The concentrate mixture comprised wheat bran (32%), corn middlings (32%), pulse hulls (10%), noug cake (25%) and salt (1%), with an average composition of 92.15% dry matter, 17.14% crude protein, 30.01% neutral detergent fibre, 17.45% acid detergent fibre and 4.61% acid detergent lignin. The cows were hand‐milked twice daily and routinely vaccinated, dewormed and treated for internal and external parasites.

### 2.3 Data Collection

#### 2.3.1 Primary Data

**Milk yield**: Data were collected from June to November 2022. This period generally corresponds to the main rainy season, extending into the early dry season in the study area. Daily milk yield was recorded for 25 consecutive weeks from sixty (Nozad et al. 2012) Boran cows stratified by lactation stage: 20 cows in early lactation, 20 cows in mid‐lactation and 20 cows in late lactation. Measurements were taken twice daily (morning and afternoon) using a calibrated jug. To account for the milk consumed by calves, calves were separated from their dams for 12 h before milking, and the amount of milk suckled was estimated by allowing the calf to suckle immediately after milking. The total daily milk yield per cow was calculated as the sum of the milk obtained during milking and the estimated milk consumed by the calf. The mean daily milk yield was calculated for each lactation stage to assess the production performance.

**Adaptive profile**: Thirty (Chang‐Fung‐Martel et al. 2021) lactating cows were selected to assess adaptive responses, including physiological, haematological and biochemical parameters of the animals. Rectal temperature (RT) (°C) and respiration rate (RR) (breaths per minute, BPM) were recorded twice weekly (morning and afternoon). Environmental temperature and humidity were simultaneously measured using dry and wet bulb thermometers at the same time. The temperature–humidity index (THI), an indicator of thermal comfort, was calculated as follows: THI = (0.81 × AT) + (RH/100) × (AT − 14.4) + 46.6, where, AT is ambient temperature (°C) and RH is the relative humidity (%) (Li et al. 2021).

**Rectal temperature**: RT was measured using a digital thermometer inserted approximately 8 cm into the rectum once a week, both in the morning and afternoon.

**Respiration rate**: RR of Boran cows was recorded twice daily (morning and afternoon) following the same schedule as RT measurements. RR was measured manually by counting flank movements for 15 s and multiplying by 4 to obtain BPM using a stopwatch without disturbing the animals (Mylostyvyi et al. 2024).

**Blood sample collection and analysis**: Blood samples were collected from thirty (Chang‐Fung‐Martel et al. 2021) lactating cows (10 per lactation stage) via jugular venipuncture into EDTA and plain tubes. Blood samples (5 mL) were collected twice during the study period for each lactation stage to capture temporal variations in haematological and biochemical parameters. Immediately after collection, the EDTA and plain tubes were gently mixed and kept in a cool box (4°C–8°C) for transportation to the laboratory. Two millilitres of blood was transferred to vacutainers without an anticoagulant for serum preparation, centrifuged at 1600 *g* for 20 min and stored at −20°C until biochemical analysis (total protein [TP], glucose [GLU], urea [U], triglycerides [TGL], total cholesterol [TC], AST and ALT). The remaining 3 mL was collected in EDTA tubes for haematological analysis (RBC, WBC, haemoglobin and PCV). Haematological parameters (HC, RBC, WBC and PCV) were analysed using standard laboratory procedures, whereas serum biochemical parameters (TP, GLU, URE, TGL, TC, AST and ALT) were measured using commercial kits following the manufacturer's instructions. Analyses were conducted at the Jimma University Teaching Hospital and Dedo General Hospital.

#### 2.3.2 Secondary Data

Secondary data on reproductive performance (AFC, CI, DO, GL and number of services per conception [NSPC]) were obtained from four years’ worth of farm records.

### 2.4 Data Analysis

Data were analysed using SPSS version 20. Descriptive statistics were computed, and the results are expressed as mean ± standard deviation (SD). The effects of lactation stage on milk yield and haematological and biochemical parameters were evaluated using analysis of variance (ANOVA), followed by least significant difference (LSD) post hoc tests for multiple comparisons. The effects of parity were assessed using independent sample *t*‐tests. Principal component analysis (PCA) was performed to identify the key variables influencing the adaptive profile of Boran dairy cattle. The suitability and reliability of the PCA were evaluated using eigenvalues (>1), varimax rotation, scree plots, the Kaiser–Meyer–Olkin (KMO) measure of sampling adequacy and Bartlett's test of sphericity. Statistical significance was set at *p* < 0.05.

The statistical model used for the analysis was: *Y*_ijk_ = *μ* + *L_i_* + *P_j_* + (*L* × *P*)_ij_ + *C_k_* + *ε*_ijk_, where *Y*_ijk_ is the observed trait (milk yield, physiological, haematological or biochemical parameter); *μ* is the overall mean; *L_i_* is the fixed effect of lactation stage (early, mid or late); *P_j_* ​ is the fixed effect of parity (*j* = 1 or 2); (*L* × *P*)_ij_ is the interaction effect between lactation stage and parity; *C_k_* is the random effect of cow (*k* = 1, 2, …, *n*); and *ε*_ijk_ is the residual error.

## 3 Results and Discussion

### 3.1 Adaptive Profile and Physiological Responses of Boran Cows

The physiological responses of Boran cows, including RR and RT, are summarized below. The mean RR was 28.83 ± 1.66 breaths/min in the morning and 37.27 ± 1.17 breaths/min in the afternoon, with an overall mean of 33.05 ± 4.48 breaths/min (Table 1). These values fell within the normal range for non‐heat‐stressed cows (26–50 breaths/min) (Lussier 2021), indicating that the cows maintained thermal equilibrium. Increases in RR are typically associated with elevated oxygen demand and heat stress, facilitating heat dissipation via respiratory evaporation (Saeed 2023).

**TABLE 1:** Respiration rate and rectal temperature of Boran cows measured in the morning and afternoon (*N* = 30).

Parameter	Morning (Mean ± SD)	Afternoon (Mean ± SD)	Overall (Mean ± SD)
RR (breaths/min)	28.83 ± 1.66	37.27 ± 1.17	33.05 ± 4.48
RT (°C)	36.30 ± 0.43	38.39 ± 0.63	37.37 ± 1.17

Abbreviations: RR, respiration rate; RT, rectal temperature.

The mean RT of Boran cows was 36.3 ± 0.43°C in the morning and 38.39 ± 0.63°C in the afternoon, with an overall mean of 37.37 ± 1.17°C (Table 1). In comparison, Lochner (2018) reported that Boran and Tuli crosses exhibited RTs around 39.5°C, intermediate between Brahman (39.0°C) and Hereford × Brahman crosses (40.0°C). While Boran cows in this study maintained normal RT ranges, other studies have shown that Boran cows can experience moderate thermal stress under higher THI conditions, with elevated skin and RTs (Giannone et al. 2023). The literature indicates that RT may rise above normal thresholds during heat stress (Fischer et al. 2012), although some studies have observed increases within normal physiological limits (Prunier et al. 2010), consistent with the present findings.

**FIGURE 2:**
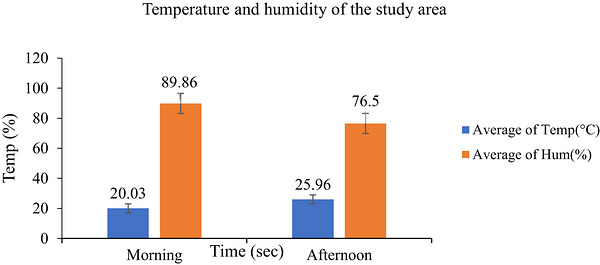
Temperature (°C) and humidity (%) of the study area measured during the morning and afternoon.

The environmental conditions during the study are shown in Figure 2. The mean ambient temperature (AT) was 20.03°C in the morning and 25.96°C in the afternoon, while the relative humidity (RH) was 89.86% and 76.50%, respectively. Although AT and RH are commonly used to assess heat stress, the THI provides a combined measure and is widely regarded as a reliable indicator of thermal stress in cattle (Habeeb et al. 2018; Chang‐Fung‐Martel et al. 2021). The THI calculated in this study was 72.41%, which is commonly used to evaluate whether animals are within the comfort or stress zones.

According to Yan et al. (2021), THI values <72 indicate the comfort zone, 72–79 indicate mild stress, 80–89 indicate moderate stress and >90 indicate severe stress in dairy cattle. Similarly, other studies have defined THI values of 73–77 and 78–89 as mild and moderate heat stress, respectively (J. Liu et al. 2019). The THI values observed in the present study indicate that the Boran cows were within the comfort zone and not exposed to heat stress. However, increasing THI levels can challenge thermoregulation, potentially reducing performance as cows attempt to maintain a stable body temperature (Lees et al. 2019).

### 3.2 Blood Biochemical Responses of Boran Cows

**FIGURE 3:**
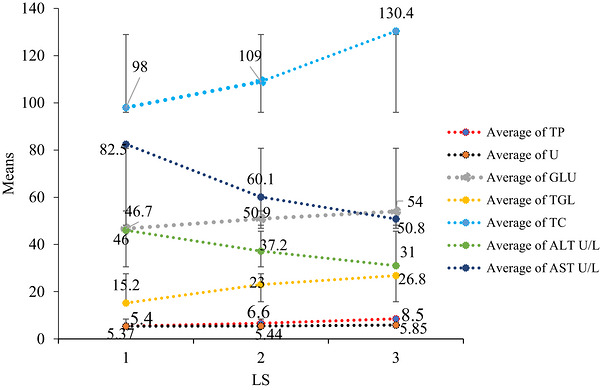
Effect of lactation stage and parity on biochemical parameters of Boran cows.

In the current study, all investigated blood biochemical parameters, including TP, U, GLU, TGL, TC, alanine aminotransferase (ALT), and aspartate aminotransferase (AST), were significantly (*p* < 0.001) influenced by the stage of lactation and parity (Table 2 and Figure 3). Effect of Lactation Stage: TP levels increased significantly (*p* < 0.001) with advancing lactation stages, showing values of 5.40, 6.60, and 8.50 g/dL at early, middle and late lactation, respectively (Table 2).

**TABLE 2:** Effect of lactation stage and parity on serum biochemical variables of Boran dairy cows (*N* = 30).

Effect	Category	*N*	TP (g/dL)	Urea (g/dL)	GLU (mg/dL)	TGL, (mg/dL)	(TC, mg/dL)	ALT (U/L)	AST (U/L)
LS	Early	10	5.40 ± 0.69^c^	5.37 ± 0.18^b^	46.70 ± 1.33^c^	15.20 ± 2.44^c^	98.00 ± 7.33^c^	46.00 ± 2.58^a^	82.50 ± 2.17^a^
	Mid	10	6.60 ± 0.69^b^	5.40 ± 0.19^b^	50.90 ± 0.87^b^	23.00 ± 1.56^b^	109.00 ± 2.70^b^	37.20 ± 1.47^b^	60.10 ± 2.60^b^
	Late	10	8.50 ± 0.85^a^	5.85 ± 0.26^a^	54.00 ± 1.05^a^	26.80 ± 1.87^a^	130.40 ± 5.66^a^	31.00 ± 1.88^c^	50.80 ± 2.53^c^
	*p*‐value	—	<0.001	<0.001	<0.001	<0.001	<0.001	<0.001	<0.001
Parity	1	15	5.73 ± 0.79^b^	5.35 ± 0.18^b^	48.13 ± 2.41^b^	18.13 ± 4.73^b^	101.33 ± 7.68^b^	42.73 ± 5.25^a^	75.40 ± 10.58^a^
	2	15	7.93 ± 1.16^a^	5.75 ± 0.25^a^	52.93 ± 1.83^a^	25.20 ± 2.90^a^	123.60 ± 11.08^a^	33.40 ± 3.85^b^	53.53 ± 4.74^b^
	*p*‐value	—	<0.001	<0.001	<0.001	<0.001	<0.001	<0.001	<0.001
Overall	—	30	6.83 ± 1.48	5.55 ± 0.30	50.53 ± 3.22	21.67 ± 5.27	112.47 ± 14.70	38.07 ± 6.56	64.47 ± 13.73

*Note*: Superscripts (a–c) within the same column indicate significant differences. Values sharing the same superscript are not significantly different (*p* > 0.05).

Abbreviations: ALT, alanine transaminase; AST, aspartate transaminase; GLU, glucose; TC, total cholesterol; TGL, triglycerides; TP, total protein; U, urea.

The highest TP was observed in late lactation, likely due to reduced milk production during this stage. These findings are in agreement with Antanaitis et al. (2021), who reported progressive TP increases in Holstein cattle (6.82, 7.05, and 7.27 g/dL). The mean TP in this study (6.83 g/dL) fell within the reference range reported for tropical dairy cattle by different scholars (4.83–8.34 g/dL; 5.6–8.1 g/dL) (Vallejo‐Timarán et al. 2020; Perumal et al. 2023; de Vasconcelos et al. 2020).

Blood U also increased with lactation stage, with values of 5.37, 5.4, and 5.85 g/dL for early, middle and late lactation, respectively (Table 2, Figure 3). The late‐lactation increase may reflect enhanced protein catabolism for maintenance and reduced milk yield, whereas the lower early lactation U level could be due to limited nutrient intake. These values are consistent with the reference ranges reported for Holstein cattle in tropical conditions (5.4–14.4 g/dL, 4.5–12.0 g/dL and 3.0–8.0 g/dL) (Cooke et al. 2020; Hudaya et al. 2020; Paiano et al. 2020).

Blood GLU levels increased significantly (*p* < 0.001) with advancing lactation (46.70, 50.9, and 54.00 mg/dL for early, middle and late lactation, respectively) (Table 2, Figure 3). Lower GLU levels during early lactation may be due to high utilization for lactose synthesis during peak milk production (H. Liu et al. 2013). The mean GLU (50.53 mg/dL) aligns with reported reference ranges for tropical Holstein cattle (45–77.4 mg/dL; 46.8–68.4 mg/dL) (Cooke et al. 2020; Hudaya et al. 2020; López et al. 2023).

TGL levels increased significantly (*p* < 0.001) from 15.20 mg/dL in early lactation to 26.80 mg/dL in late lactation, while TC also increased from 98.00 to 130.40 mg/dL (Table 2, Figure 3). Lower early lactation values may result from high milk yield and metabolic stress (Marett et al. 2019). The activity of ALT decreased from early (46.00 U/L) to late lactation (31.00 U/L), with a mean of 38.07 U/L, within the normal reference range of 20–45 U/L. (Bogolyubova et al. 2021).

AST also decreased from 82.50 U/L in early lactation to 50.80 U/L in late lactation, possibly due to physiological stress during the metabolic transition from pregnancy to lactation (Mohamed et al. 2019). The mean AST (64.47 U/L) was within the reference range for Holstein cows (19.21–84.97 U/L) (Porosnicu et al. 2024). Effect of Parity: TP increased with parity, with Parity 1 cows showing 5.73 ± 0.79 g/dL and Parity 2 cows showing 7.93 ± 1.16 g/dL (Table 2, Figure 3). Similarly, blood GLU levels increased from 48.13 mg/dL (Parity 1) to 52.93 mg/dl (Parity 2).

TGL levels increased from 18.13 ± 4.73 to 25.20 ± 2.90 mg/dL, and TC levels were higher in Parity 2 cows. Blood U was slightly higher in Parity 2 cows (5.75 g/dL) than in Parity 1 cows (5.35 g/dL) (Table 2, Figure 3). ALT and AST levels decreased with parity, consistent with the findings in Holstein Friesian cows (Porosnicu et al. 2024; Yehia et al. 2020). Overall, lactation stage and parity significantly influenced the biochemical profiles of Boran cows, reflecting metabolic adaptations to milk production, nutrient availability and physiological status.

### 3.3 Haematological Parameters

**FIGURE 4:**
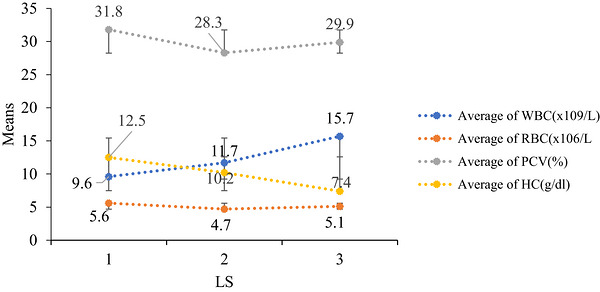
Effect of lactation stage on haematological parameters of Boran cows.

The effects of lactation stage and parity on the haematological values of lactating Boran cattle in the current study are presented below. The mean values of hemoglobin concentration (HC), white blood cell count (WBC) and packed cell volume (PCV) were significantly influenced by the stage of lactation (*p* < 0.001), whereas the red blood cell count (RBC) did not differ significantly across lactation stages (*p* > 0.05) (Table 3, Figure 4). In this study, HC decreased progressively across lactation stages, with mean values of 12.50, 10.20 and 7.40 g/dL at early, mid and late lactation, respectively.

**TABLE 3:** Effect of lactation stage and parity on haematological parameters of Boran cows (*N* = 30).

Effect	Category	*N*	WBC (×10^9^/L)	RBC (×10^6^/L)	PCV (%)	Hb (g/dL)
LS	Early	10	9.60 ± 1.50^c^	5.60 ± 0.90	31.80 ± 1.03^a^	12.50 ± 1.50^a^
	Mid	10	11.70 ± 1.25^b^	4.70 ± 0.90	28.30 ± 0.60^c^	10.20 ± 0.91^b^
	Late	10	15.70 ± 0.80^a^	5.10 ± 0.50	29.90 ± 0.80^b^	7.40 ± 1.10^c^
	*p*‐value	—	<0.001	0.77	<0.001	<0.001
Parity	1	15	10.20 ± 1.61^b^	5.33 ± 0.97	30.73 ± 1.79^a^	11.73 ± 1.79^a^
	2	15	14.47 ± 2.06^a^	4.93 ± 0.79	29.27 ± 1.22^b^	8.33 ± 1.71^b^
	*p*‐value	—	<0.001	0.23	0.015	<0.001
Overall	—	30	12.33 ± 2.83	5.13 ± 0.90	30.00 ± 1.68	10.03 ± 2.44

*Note: N* is the total number of observations; abc‐ in the same column indicates significancy *p* < 0.001;

Abbreviations: HC, haemoglobin concentration; PCV, packed cell volume; RBC, red blood cell; WBC, white blood cell.

The mean RBC count in this study was 5.13 × 10^6^/L, which falls within the reference range for dairy cattle (5–10 × 10^6^/L) reported by Chen et al. (2022). WBC increased significantly with advancing lactation, with mean values of 9.60, 11.70 and 15.70 × 10^9^/L at the early, mid and late stages, respectively. These values were within the reference range for healthy dairy cows (5.04–16.66 × 10^9^/L) reported by Chen et al. (2022).

The PCV values were 31.80%, 28.30% and 29.9% for the early, mid and late lactation stages, respectively. The lower PCV observed at mid‐lactation may correspond to the slightly lower RBC count at this stage. All PCV values fell within the normal reference range for cattle (24%–46%) (Chen et al. 2022; Radkowska and Herbut 2014).

Haematological parameters, including WBC, PCV and HC, were significantly influenced by parity (*p* < 0.001). The WBC count was significantly higher in cows of Parity 2 (14.47 × 10^9^/L) than in cows of Parity 1 (10.20 × 10^9^/L), suggesting enhanced immune activity in cows of higher parity (Table 3). Similar trends were reported by Chen et al. (2022), who recorded WBC values of 10.3 ± 3.17 and 11.23 ± 3.89 × 10^9^/L for Parity 1 and Parity 2 Holstein–Friesian cows, respectively. Conversely, RBC count decreased with increasing parity, with Parity 1 and Parity 2 cows showing 5.33 ± 0.97 and 4.93 ± 0.79 × 10^6^/L, respectively.

The reduction in RBC in Parity 2 cows may be due to higher production demands, leading to lower blood oxygen content or deficiencies in iron and vitamins during lactation (Zeng et al. 2023). Chen et al. (2022) also reported decreased RBC in Parity 2 cows (5.82 ± 0.63 × 10^12^/L) compared to Parity 1 cows (6.54 ± 0.59 × 10^12^/L). PCV decreased significantly in Parity 2 cows (29.27 ± 1.22%) compared to Parity 1 cows (30.73 ± 1.79%). Similarly, HC declined with parity, recording 11.73 ± 1.79 g/dL in Parity 1 and 8.33 ± 1.71 g/dL in Parity 2 cows. Vallejo‐Timarán et al. (2020) also reported a significant decrease in HC with increasing parity in grazing cows in tropical Colombia. This decline may be attributed to the higher productive capacity of Parity 2 cows, which can lead to blood loss during lactation and result in lower RBC levels and anaemia (Yehia et al. 2020). Overall, HC, RBC and PCV were critical indicators of oxygen transport capacity and general health status in lactating Boran cattle, whereas WBC reflected immune competence.

### 3.4 PCA Analysis of Blood Parameters

**FIGURE 5:**
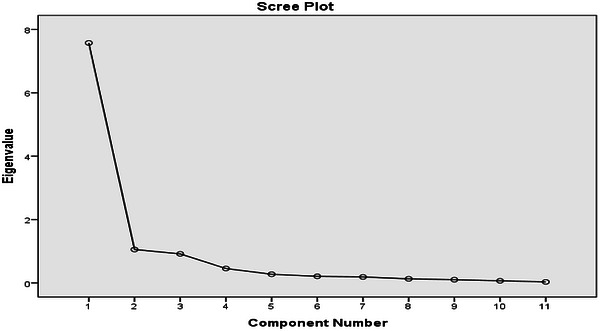
Scree plot of blood parameters in lactating Boran cows showing the eigenvalues of principal components.

The adequacy of the sample for factor analysis in this study was assessed using the KMO measure, which was 0.894 (Table 4, Figure 5), classified as ‘Meritorious’ (Zhang et al. 2024). A KMO value greater than 0.50 indicates that the data are suitable for factorial analysis (Hadi et al. 2016). Furthermore, Bartlett's test of sphericity was significant (*p* < 0.001), indicating that the correlations between the variables were sufficiently large for PCA.

**TABLE 4:** Eigenvalues and explained variance of principal components derived from principal component analysis.

Component	Eigenvalue	Variance explained (%)	Cumulative variance (%)
1	7.575	68.86	68.86
2	1.055	9.59	78.45
3	0.917	8.33	86.78
4	0.455	4.13	90.91
5	0.273	2.49	93.40
6	0.209	1.90	95.30
7	0.189	1.72	97.02
8	0.128	1.17	98.19
9	0.101	0.92	99.11
10	0.067	0.61	99.71
11	0.032	0.29	100.00

The results of all blood variables were analysed and ranked according to their eigenvalues and the proportion of variance explained, providing insight into their relative importance in the physiological responses of lactating Borana cows (Table 4, Figure 5). Eigenvectors reflect the weight of each variable in the components, with higher eigenvalues indicating a greater influence (Chun et al. 2016). PCA of the blood variables in this study showed that TP and U were the most influential parameters, while other variables, including GLU, TGL, TC, AST, ALT, HC, RBC, WBC and PCV, contributed to smaller portions of the variance.

According to Ruscio and Roche (2012), components with eigenvalues greater than 1 are retained as being significant. In this study, two components (TP and U) had eigenvalues exceeding 1 and together explained 78.447% of the total variance. Variables with smaller eigenvalues contributed minimally and were largely uncorrelated with each other. The predominance of TP and U in explaining the variation in blood profiles reflects their importance in the metabolic and nutritional status of lactating Boran cows (Andjelić et al. 2022).

Changes in TP and U levels are closely linked to reproductive cycles, lactation and the overall metabolic state. Fluctuations in these parameters throughout the day may result from variations in protein and energy intake, utilization by the mammary glands or differences in nutrient absorption (Ceciliani et al. 2018; Rezaei et al. 2016). U, while not fully utilized for milk production, is a key metabolite derived from dietary protein and tissue turnover, reflecting protein catabolism both within the rumen (by microbial activity) and systemic metabolism (Marín‐García et al. 2022). Similarly, TP levels are influenced by dietary intake and physiological demand. These factors, in turn, affect other haematological and biochemical parameters, such as GLU, RBC, WBC and HC (Weiner et al. 2015; Tedeschi et al. 2015).

Nozad et al. (2012) reported that blood U and TP levels are sensitive to management and nutritional practices. Understanding these variations can help producers optimize nutrient density, adjust diets appropriately and improve productivity while ensuring that the physiological and metabolic needs of cows for growth, lactation and reproduction are met. Thus, TP and U were the primary contributors to the variability in the blood profiles of lactating Boran cows, highlighting their significance as indicators of metabolic status and nutritional adequacy.

The scree plot also supported the retention of two components. According to Ledesma et al. (2015), the cutoff point for selecting factors is determined at the point of inflection of the curve, where the slope changes markedly. In this study, the scree plot exhibited an ‘elbow’ at the second component, indicating that two factors should be retained for further analysis (Table 4, Figure 5).

### 3.5 Productive and Reproductive Performances

#### 3.5.1 Reproductive Performance

The overall AFC, CI, DO, GL and NSPC were 51.63 months, 19.07 months, 338.05 days, 276.50 days and 1.6, respectively (Table 5). The AFC (51.63 months) reported in this study was lower than that reported in previous studies on Ethiopian Boran cattle (57.6 and 56 months, respectively) (Mengistu et al. 2016). This improvement may be attributed to better management, feeding systems and favourable environmental conditions (Crowe et al. 2018).

**TABLE 5:** Descriptive statistics of reproductive performance parameters of Boran dairy cows (*N* = 60).

Parameter	Unit	Mean ± SD
AFC	months	51.63 ± 1.82
CI	months	19.07 ± 1.90
DO	days	338.05 ± 21.70
GL	days	276.50 ± 2.10
NSPC	—	1.60 ± 0.22

Abbreviations: AFC, age at first calving; CI, calving interval; DO, days open; GL, gestation length; NSPC, number of services per conception.

The mean CI of 19.07 months falls within the reported range for zebu cattle (12.2–26.6 months) (Segura‐Correa et al. 2017). Shorter CI (17.91 ± 1.01 months) and AFC (48.39 ± 1.41 months) were reported by Woldeyohannes et al. (2024) for Boran cows at Yabello, while longer CI (20.47 months) and AFC (57.6 months) were reported by Mandefro et al. (2017) for the same breed. Differences in CI and AFC are likely due to the management, feed availability, age and parity of the cows. The mean DO of 338.05 days in this study was lower than the 622.6 days reported by Bekele and Husiena (2018), indicating improved reproductive efficiency under the current management system in this study.

The mean NSPC was 1.6, which aligns with the optimal fertility range (1.6–1.8) suggested by Cielava et al. (2017). Effective heat detection and timely insemination likely contribute to this performance (Khan et al. 2023).

#### 3.5.2 Productive Performance

##### 3.5.2.1 Average Daily Milk Yield as Affected by Lactation Stage

The average daily milk yield of Boran cows varied across lactation stages, with mean values of 2.43, 2.12 and 1.72 L/day during the early, mid and late lactation stages, respectively. Milk yield was significantly lower (*p* < 0.001) in the late lactation stage than in the early and mid‐lactation periods (Table 6). This pattern is consistent with the findings of Vijayakumar et al. (2017), who reported that milk yield typically increases during the first 90 days of lactation, remains relatively stable for a short duration, and subsequently declines as lactation progresses, likely due to changes in udder development and the activity of mammary secretory cells.

**TABLE 6:** Effect of lactation stage and parity on milk yield (L/day) of Boran dairy breeds in the study area (*N* = 60).

Factor	Level	*N*	Milk yield (Mean ± SD)
Overall		60	2.09 ± 0.62
Lactation stage	Early	20	2.43 ± 0.62^a^
	Mid	20	2.12 ± 0.58^a^
	Late	20	1.72 ± 0.45^c^
	*p*‐value		<0.05
Parity	1	30	1.71 ± 0.46^b^
	2	30	2.47 ± 0.52^a^
	*p*‐value		<0.05

*Note*: Different superscripts (^a–c^) within the same variable indicate significant differences (*p* < 0.05).

The overall mean daily milk yield recorded in the present study (2.09 L/day) exceeded the values reported by Abegaz (2022) for indigenous cows (1.48 and 1.30 L/day) but was lower than the range of 2.28–3.92 L/day reported by Abebe et al. (2017). Similarly, Jembere et al. (2021) observed a mean yield of 2.21 ± 0.42 L/day for Boran cows at the Holeta Agricultural Research Center, Ethiopia. The observed differences in milk yield among studies may be attributed to variations in feed quality and availability, management practices and environmental conditions of the animals.

#### 3.5.3 Milk Yield as Affected by Parity

The average daily milk yield for Parity 1 and Parity 2 cows was 1.71 and 2.47 L/day, respectively, indicating an approximate 30% increase in parity 2 cows (Table 6). First‐parity cows are still growing, with developing mammary glands and low milk production capacities. Hoka et al. (2019) reported an increasing trend in milk yield with parity in local cows (1.5, 2.1, 2.5, 3.5 and 1.5 L at Parities 1–5, respectively). Similarly, Kebede (2018) observed higher milk production in 2nd‐parity Holstein cows (15.88 L) than in first‐parity cows (14.45 L). Dauda et al. (2023) also reported increased milk yield in Parity 2 Boran cows (2.25 L/day) compared to parity 1 (1.95 L/day). The current results indicate that both lactation stage and parity significantly affected milk production (*p* < 0.05) (Table 6).

## 4 Conclusion and Recommendations

The present study demonstrated that the daily milk yield of Boran cows was significantly influenced by lactation stage and parity, with higher production during early and mid‐lactation and in Parity 2 cows compared with Parity 1 cows. PCA identified TP and U as the main contributors to the variation in blood biochemical profiles, and lactation stage and parity significantly affected haematological and biochemical parameters. All measured values remained within the normal physiological reference ranges, indicating good metabolic stability and adaptation of the cows to the production environment. Milk yield and reproductive performance were comparable to those of other indigenous cattle breeds, supporting the suitability of Boran cattle for dairy production under similar conditions in Ethiopia. To further optimize productive and reproductive performance, improved nutritional management, healthcare and environmental management should be emphasized, and carefully planned crossbreeding programs should be considered without compromising adaptive capacity. As seasonal effects were not analysed separately, the results represent average performance across the data collection period, and future studies should incorporate seasonal comparisons.

## Author Contributions

**Ephrem Worku Nigatu**: conceptualization, investigation, writing – original draft, methodology, validation, visualization, writing – review and editing, software, formal analysis, data curation.

## Funding

The author has nothing to report.

## Ethics Statement

All procedures involving animals, including milking, blood sampling and calf separation, were conducted in accordance with international guidelines for the care and use of agricultural animals in research. Measures were taken to minimize stress and discomfort to the animals throughout the study period.

## Conflicts of Interest

The author declares no conflicts of interest.

## Data Availability

Data supporting the findings of this study are available upon reasonable request by contacting the corresponding author

## References

[vms371053-bib-0001] Abdurehman, A. 2019. “Physiological and Anatomical Adaptation Characteristics of Borana Cattle to Pastoral Lowland Environments.” Asian Journal of Biological Sciences 12: 364–372.

[vms371053-bib-0002] Abebe, B. , Y. Zelalem , E. Mitiku , Y. Mohammed , and A. Getenet . 2017. “Socio‐Economic Characteristics of Dairy Production in Ethiopia.” Journal of Veterinary Medicine and Animal Health 9, no. 8: 193–203.

[vms371053-bib-0003] Abegaz, S. B. 2022. “Milk Production Status and Associated Factors Among Indigenous Dairy Cows in Raya Kobo District, Northeastern Ethiopia.” Veterinary Medicine and Science 8, no. 2: 852–863. 35080133 10.1002/vms3.740 PMC8959299

[vms371053-bib-0004] Andjelić, B. , R. Djoković , M. Cincović , et al. 2022. “Milk and Blood Biochemical Parameters During Lactation.” Metabolites 12, no. 8: 733. 36005606 10.3390/metabo12080733 PMC9412388

[vms371053-bib-0005] Antanaitis, R. , V. Juozaitienė , V. Jonike , et al. 2021. “Relationship Between Temperament, Lactation Stage, Productivity and Milk Composition in Dairy Cows.” Animals 11, no. 7: 1840. 34206163 10.3390/ani11071840 PMC8300410

[vms371053-bib-0006] Asfaw, Y. , R. Begna , and W. Masho . 2023. “Evaluation of Breeding Objectives, Breeding Practices and Reproductive Performance of Indigenous Dairy Cows in Selected Districts of Kaffa Zone, Southwestern Ethiopia.” Veterinary Medicine and Science 9, no. 6: 2820–2834. 37728180 10.1002/vms3.1267 PMC10650342

[vms371053-bib-0007] Assefa, A. , A. Hailu , A. Mustefa , A. Melak , and T. Getachew . 2021. “Characterization, Conservation and Sustainable Utilization of Ethiopian Animal Genetic Resources: Status, Challenges and Opportunities.” International Journal of Social Science Studies 3, no. 1: 230–241.

[vms371053-bib-0008] Barsila, S. R. 2025. “Blood Hematologic and Biochemical Values of High‐Altitude Pastoral Animals.” Journal of Agriculture and Forestry University 6, no. 1: 199–211.

[vms371053-bib-0009] Bayssa, M. , S. Yigrem , S. Betsha , and A. Tolera . 2021. “Production, Reproduction and Adaptation Characteristics of Boran Cattle Under Changing Climate: A Systematic Review and Meta‐Analysis.” PLoS One 16, no. 5: e0244836. 34048433 10.1371/journal.pone.0244836 PMC8162631

[vms371053-bib-0010] Bekele, B. , and B. Husiena . 2018. “Performance Evaluation of Borana Cattle Under Rangeland Conditions.” In Proceedings of the Regional Review Workshop on Completed Research Activities 12: 12.

[vms371053-bib-0011] Bogolyubova, N. V. , V. N. Romanov , and V. A. Bagirov . 2021. “Metabolic Profile of Cows During Late Dry Period and Early Lactation.” Russian Agricultural Sciences 47, no. 2: 155–160.

[vms371053-bib-0012] Ceciliani, F. , C. Lecchi , C. Urh , and H. Sauerwein . 2018. “Proteomics and Metabolomics of Metabolic Challenges in Transition Dairy Cows.” Journal of Proteomics 178: 92–106. 29055723 10.1016/j.jprot.2017.10.010

[vms371053-bib-0013] Chang‐Fung‐Martel, J. , M. T. Harrison , J. N. Brown , R. Rawnsley , A. P. Smith , and H. Meinke . 2021. “Negative Relationship Between Dry Matter Intake and Temperature–Humidity Index in Cattle: A Global Meta‐Analysis.” International Journal of Biometeorology 65, no. 12: 2099–2109. 34283273 10.1007/s00484-021-02167-0 PMC8566424

[vms371053-bib-0014] Chen, H. , B. Yu , C. Liu , et al. 2022. “Hematology Reference Intervals for Holstein Cows in Southern China.” Veterinary Science 9, no. 10: 565. 10.3390/vetsci9100565 PMC9611909 36288178

[vms371053-bib-0015] Chun, Y. , D. A. Griffith , M. Lee , and P. Sinha . 2016. “Eigenvector Selection With Stepwise Regression.” Journal of Geographical Systems 18, no. 1: 67–85.

[vms371053-bib-0016] Cielava, L. , D. Jonkus , and L. Paura . 2017. “Services Per Conception and Dairy Cow Performance.” Research in Rural Development 2: 67–73.

[vms371053-bib-0017] Cooke, R. F. , R. C. Cardoso , R. L. A. Cerri , et al. 2020. “Cattle Adapted to Tropical and Subtropical Environments: Genetic and Reproductive Considerations.” Journal of Animal Science 98, no. 2: skaa015. 31955201 10.1093/jas/skaa015 PMC7032034

[vms371053-bib-0018] Crowe, M. A. , M. Hostens , and G. Opsomer . 2018. “Reproductive Management in Dairy Cows: The Future.” Irish Veterinary Journal 71, no. 1: 1. 29321918 10.1186/s13620-017-0112-y PMC5759237

[vms371053-bib-0019] Dauda, A. , Y. Idi , and D. Jibrin . 2023. “Effect of Parity on Milk Proximate and Mineral Composition in Cattle.” FUDMA Journal of Agriculture and Agricultural Technology 9, no. 2: 87–91.

[vms371053-bib-0020] Dekebo, D. , and I. A. Kebede . 2023. “Review on Dairy Cattle Production in Ethiopia.” Mathews Journal of Veterinary Science 7, no. 4: 1–7.

[vms371053-bib-0021] Denbarga, Y. , B. Woldegebriel , and D. Shiferaw . 2012. “Reproductive Performance of Boran Cows at Tatesa Breeding Center.” Advances in Biological Regulation 6: 101–105.

[vms371053-bib-0022] de Vasconcelos, A. M. , C. C. de Albuquerque , J. F. de Carvalho , et al. 2020. “Adaptive Profile of Dairy Cows in a Tropical Region.” International Journal of Biometeorology 64, no. 1: 105–113. 31485808 10.1007/s00484-019-01797-9

[vms371053-bib-0023] Endris, M. 2017. “Milk Production and Reproductive Performance of Dairy Cattle in Ethiopia: A Review.” Online Journal of Animal and Feed Research 7: 154–160.

[vms371053-bib-0024] Fischer, E. M. , K. W. Oleson , and D. M. Lawrence . 2012. “Contrasting Urban and Rural Heat Stress Responses to Climate Change.” Geophysical Research Letters 39, no. 3: L03705.

[vms371053-bib-0025] Galina, C. S. , and M. Geffroy . 2023. “Dual‐Purpose Cattle Raised in Tropical Conditions: Shortcomings in Productive and Reproductive Performance.” Animals 13, no. 13: 2224. 37444022 10.3390/ani13132224 PMC10339982

[vms371053-bib-0026] Getahun, K. 2022. “Estimates of Breed Additive, Heterosis and Epistasis Effects on Milk Production and Reproductive Traits of Borena and Holstein‐Friesian Crosses in Ethiopia.” Multidisciplinary Science Journal 4, no. 3: 2022013.

[vms371053-bib-0027] Giannone, C. , M. Bovo , M. Ceccarelli , D. Torreggiani , and P. Tassinari . 2023. “Heat Stress‐Induced Responses in Dairy Cattle: A Review.” Animals 13, no. 22: 3451. 38003069 10.3390/ani13223451 PMC10668733

[vms371053-bib-0028] Habeeb, A. A. , A. E. Gad , and M. A. Atta . 2018. “Temperature–Humidity Indices as Indicators of Heat Stress Related to Production and Reproduction of Farm Animals.” International Journal of Biotechnology and Recent Advances 1, no. 1: 35–50.

[vms371053-bib-0029] Hadi, N. U. , N. Abdullah , and I. Sentosa . 2016. “An Easy Approach to Exploratory Factor Analysis.” Journal of Educational and Social Research 6, no. 1: 215–223.

[vms371053-bib-0030] Hoka, A. I. , M. Gicheru , and S. Otieno . 2019. “Effect of Cow Parity on Milk Production and Reproduction.” Experimental Animals 9: 7176.

[vms371053-bib-0031] Hudaya, M. F. , P. I. Sitaresmi , C. T. Noviandi , B. P. Widyobroto , and D. T. Widayati . 2020. “Behaviour and Blood Profile of Friesian‐Holstein Cows in Yogyakarta, Indonesia.” Journal of Animal Behaviour and Biometeorology 8, no. 4: 244–249.

[vms371053-bib-0032] Hunde, D. , and Y. Tadese . 2020. “Genetic Distance and Differentiation Among Cattle Breeds in Ethiopia: A Review.” Ethiopian Journal of Animal Production 20, no. 1: 80–88.

[vms371053-bib-0033] Jembere, T. , U. Galmessa , M. Tadese , M. Shumiye , and F. Wedajo . 2021. “The Influence of Different Factors on Reproductive and Productive Performances of Dairy Farm: The Case of Holeta Agricultural Research Center.” ESAP Proceedings, 167.

[vms371053-bib-0034] Karthik, D. , J. Suresh , Y. R. Reddy , et al. 2021. “Adaptive Profiles of Nellore Sheep Across Farming Systems and Seasons.” Heliyon 7, no. 5: e07053. 34136691 10.1016/j.heliyon.2021.e07117 PMC8176311

[vms371053-bib-0035] Kebede, E. 2018. “Effect of Cattle Breed on Milk Composition.” Ethiopian Journal of Agricultural Sciences 28, no. 2: 53–64.

[vms371053-bib-0036] Khan, I. , A. Mesalam , Y. S. Heo , S. H. Lee , G. Nabi , and I. K. Kong . 2023. “Heat Stress and Reproduction in Cattle.” Animals 13, no. 14: 2359. 37508136 10.3390/ani13142359 PMC10376617

[vms371053-bib-0037] Ledesma, R. D. , P. Valero‐Mora , and G. Macbeth . 2015. “Scree Test and Number of Factors.” Spanish Journal of Psychology 18: E11. 26055575 10.1017/sjp.2015.13

[vms371053-bib-0038] Lees, A. M. , V. Sejian , A. L. Wallage , et al. 2019. “The Impact of Heat Load on Cattle.” Animals 9, no. 6: 322. 31174286 10.3390/ani9060322 PMC6616461

[vms371053-bib-0039] Li, M. , F. U. Hassan , Z. Tang , et al. 2021. “Physiological, Oxidative and Metabolic Responses of Lactating Water Buffaloes to Tropical Climate of South China.” Veterinary Medicine and Science 7, no. 5: 1696–1706. 34273254 10.1002/vms3.570 PMC8464237

[vms371053-bib-0040] Liu, J. , L. Li , X. Chen , Y. Lu , and D. Wang . 2019. “Effects of Heat Stress on Body Temperature, Milk Production and Reproduction in Dairy Cows: A Review.” Asian‐Australasian Journal of Animal Sciences 32, no. 9: 1332–1340. 30744345 10.5713/ajas.18.0743 PMC6722315

[vms371053-bib-0041] Liu, H. , K. Zhao , and J. Liu . 2013. “Effects of Glucose Availability on Genes Involved in Milk Synthesis in Bovine Mammary Epithelial Cells.” PLoS One 8, no. 6: e66092. 23799073 10.1371/journal.pone.0066092 PMC3682949

[vms371053-bib-0042] Lochner, D. 2018. “Phenotypic and Genetic Characterization of South African Boran Cattle.” PhD diss., University of Pretoria.

[vms371053-bib-0043] López, C. , V. Hincapié , and J. U. Carmona . 2023. “Comparison of Two Methods for Blood Glucose Measurement in Tropical Highland Dairy Cows.” Animals 13, no. 22: 3536. 38003153 10.3390/ani13223536 PMC10668638

[vms371053-bib-0044] Lussier, C. 2021. “Impacts of Summer Outdoor Access on Heat Stress and Panting Behaviour of Lactating Holstein Cows Housed in Tie‐Stall.” Master's thesis, McGill University.

[vms371053-bib-0045] Mandefro, A. , G. Duguma , T. Mirkena , and H. Dadi . 2017. “Alternative Breeding Plans for Ethiopian Indigenous Cattle.” Livestock Science 205: 122–128.

[vms371053-bib-0046] Marchioretto, P. V. , R. C. Rabel , C. A. Allen , M. M. Ole‐Neselle , and M. B. Wheeler . 2023. “Development of Genetically Improved Tropical‐Adapted Dairy Cattle.” Animal Frontiers 13, no. 5: 7–15. 10.1093/af/vfad050 PMC10575304 37841756

[vms371053-bib-0047] Marett, L. C. , M. J. Auldist , W. J. Wales , K. L. Macmillan , F. R. Dunshea , and B. J. Leury . 2019. “Metabolic Responses of Dairy Cows During Extended Lactation.” Journal of Dairy Science 102, no. 5: 4590–4605. 30827560 10.3168/jds.2018-15513

[vms371053-bib-0048] Marín‐García, P. J. , L. Llobat , M. C. López‐Luján , M. Cambra‐López , E. Blas , and J. J. Pascual . 2022. “Urea Nitrogen Metabolite and Ideal Protein Concept.” Animals 12, no. 18: 2344. 36139206 10.3390/ani12182344 PMC9495106

[vms371053-bib-0049] Mekonnen, A. , A. Haile , T. Dessie , Y. Mekasha , and G. Duguma . 2022. “Evaluation of Alternative Breeding Strategies for Horro Cattle Herds in Western Oromia, Ethiopia.” Journal of Science, Technology and Arts Research 11, no. 3: 19–30.

[vms371053-bib-0050] Mengistu, D. W. , K. A. Wondimagegn , and M. H. Demisash . 2016. “Reproductive Performance of Holstein Friesian and Boran Crosses in Ethiopia.” Iranian Journal of Applied Animal Science 6, no. 4: 823–830.

[vms371053-bib-0051] Mohamed, E. A. , S. A. Abd El‐Rahim , Y. A. E. Mahmoud , and A. Mohamed . 2019. “Metabolic Profiles During Lactation in Cows.” Assiut Veterinary Medical Journal 65, no. 161: 263–269.

[vms371053-bib-0052] Mollong, E. , M. Lébri , C. Marie‐Magdeleine , S. M. Lagou , M. Naves , and J. C. Bambou . 2025. “Sustainable Management of Tick Infestations in Cattle: A Tropical Perspective.” Parasites Vectors 18, no. 1: 62. 39980048 10.1186/s13071-025-06684-4 PMC11841269

[vms371053-bib-0053] Mylostyvyi, R. , V. Sejian , J. B. Souza‐Junior , et al. 2024. “Digitalisation Opportunities for Livestock Welfare Monitoring With a Focus on Heat Stress.” Multidisciplinary Reviews 7, no. 12: 2024300.

[vms371053-bib-0054] Nozad, S. , A. G. Ramin , G. Moghadam , S. Asri‐Rezaei , A. Babapour , and S. Ramin . 2012. “Blood Metabolites and Milk Quality in Holstein Cows.” Veterinary Research Forum 3, no. 1: 55–60. 25653747 PMC4312820

[vms371053-bib-0055] Paiano, R. B. , D. B. Birgel , J. Bonilla , and E. H. Birgel Jr . 2020. “Biochemical Profile of Dairy Cows With Metabolic Diseases Under Tropical Conditions.” Reproduction in Domestic Animals = Zuchthygiene 55, no. 9: 1219–1228. 32634252 10.1111/rda.13768

[vms371053-bib-0056] Pedlar, C. R. , J. Newell , and N. A. Lewis . 2019. “Blood Biomarker Profiling for High‐Performance Physiology and Nutrition.” Sports Medicine 49, no. S2: 185–198. 10.1007/s40279-019-01158-x PMC6901403 31691931

[vms371053-bib-0057] Perumal, P. , A. K. De , D. Bhattacharya , and E. B. Chakurkar . 2023. “Lactation Stage‐Related Changes in Hematological, Biochemical and Oxidative Stress Markers in Crossbred Cows.” Tropical Animal Health and Production 55, no. 2: 131. 36964324 10.1007/s11250-023-03544-0

[vms371053-bib-0058] Porosnicu, I. , L. I. Ailincai , and A. S. Neculai‐Valeanu , et al. 2024. “Health Status of Dairy Cows Assessed by Serum Biochemical Parameters.” Scientific Papers Animal Science and Biotechnologies 57, no. 2: 63–68.

[vms371053-bib-0059] Prunier, A. , M. Heinonen , and H. Quesnel . 2010. “High Physiological Demands in Intensively Raised Pigs: Impact on Health and Welfare.” Animal 4, no. 6: 886–898. 22444261 10.1017/S175173111000008X

[vms371053-bib-0060] Radkowska, I. , and E. Herbut . 2014. “Hematological and Biochemical Blood Parameters in Dairy Cows Depending on Management System.” Animal Science Papers and Reports 32, no. 4: 317–325.

[vms371053-bib-0061] Rezaei, R. , Z. Wu , Y. Hou , F. W. Bazer , and G. Wu . 2016. “Amino Acids and Mammary Gland Development.” Journal of Animal Science and Biotechnology 7: 20. 27042295 10.1186/s40104-016-0078-8 PMC4818943

[vms371053-bib-0062] Ruscio, J. , and B. Roche . 2012. “Determining the Number of Factors in Exploratory Factor Analysis.” Psychological Assessment 24, no. 2: 282–292. 21966933 10.1037/a0025697

[vms371053-bib-0063] Saeed, O. A. 2023. “Effects of Heat Stress on Ruminant Physiological Changes in Dry Arid Regions: A Review.” Large Animal Review 29, no. 6: 271–278.

[vms371053-bib-0064] Segura‐Correa, J. C. , J. G. Magaña‐Monforte , J. R. Aké‐López , V. M. Segura‐Correa , J. A. Hinojosa‐Cuellar , and M. M. Osorio‐Arce . 2017. “Breed and Environment Effects on Zebu Cattle Performance.” Tropical and Subtropical Agroecosystems 20, no. 2: 297–305.

[vms371053-bib-0065] Tedeschi, L. O. , D. G. Fox , M. A. Fonseca , and L. F. Cavalcanti . 2015. “Models of Protein and Amino Acid Requirements for Cattle.” Revista Brasileira de Zootecnia 44: 109–132.

[vms371053-bib-0066] Vallejo‐Timarán, D. , J. Montoya‐Zuluaga , V. Castillo‐Vanegas , and J. Maldonado‐Estrada . 2020. “Parity and Season Effects on Hematological, Biochemical and Milk Parameters in Postpartum Dairy Cows.” Heliyon 6, no. 5: e03941. 32490249 10.1016/j.heliyon.2020.e04049 PMC7256465

[vms371053-bib-0067] Vanvanhossou, S. F. , L. H. Dossa , and S. König . 2021. “Sustainable Management of Animal Genetic Resources to Improve Low‐Input Livestock Production: Insights Into Local Beninese Cattle Populations.” Sustainability 13, no. 17: 9874.

[vms371053-bib-0068] Vijayakumar, M. , J. H. Park , K. S. Ki , et al. 2017. “Effects of Lactation Factors on Milk Yield in Holstein Cows.” Asian‐Australasian Journal of Animal Sciences 30, no. 8: 1093–1101. 28423887 10.5713/ajas.16.0882 PMC5494482

[vms371053-bib-0069] Weiner, I. D. , W. E. Mitch , and J. M. Sands . 2015. “Urea and Ammonia Metabolism.” Clinical Journal of the American Society of Nephrology 10, no. 8: 1444–1458. 25078422 10.2215/CJN.10311013 PMC4527031

[vms371053-bib-0070] Welay, G. M. , D. G. Tedla , G. G. Teklu , et al. 2018. “A Preliminary Survey of Major Diseases of Ruminants and Management Practices in Western Tigray Province, Northern Ethiopia.” BMC Veterinary Research 14, no. 1: 293. 30257672 10.1186/s12917-018-1621-y PMC6158858

[vms371053-bib-0071] Woldeyohannes, T. , S. Betsha , and A. Melesse . 2024. “Genetic Improvement of Indigenous Cattle Breeds Under Climate Change.” Veterinary Integrative Sciences 22, no. 1: 231–250.

[vms371053-bib-0072] Yan, G. , H. Li , and Z. Shi . 2021. “Evaluation of Thermal Indices as Indicators of Heat Stress in Dairy Cows in a Temperate Climate.” Animals 11, no. 8: 2459. 34438916 10.3390/ani11082459 PMC8388788

[vms371053-bib-0073] Yehia, S. G. , E. S. Ramadan , E. A. Megahed , and N. Y. Salem . 2020. “Effect of Parity on Metabolic and Oxidative Stress Profiles in Holstein Cows.” Veterinary World 13, no. 12: 2780–2786. 33487998 10.14202/vetworld.2020.2780-2786 PMC7811534

[vms371053-bib-0074] Zeng, J. , J. Cai , D. Wang , H. Liu , H. Sun , and J. Liu . 2023. “Heat Stress Affects Dairy Cow Health via Blood Oxygen Availability.” Journal of Animal Science and Biotechnology 14, no. 1: 112. 37658441 10.1186/s40104-023-00915-3 PMC10474781

[vms371053-bib-0075] Zhang, Z. , T. Sangsawang , K. Vipahasna , and M. Pigultong . 2024. “Integrating IPA and KMO in Applied Talent Cultivation.” Journal of Applied Data Sciences 5, no. 1: 256–267.

